# Criação e Implementação de um Banco de Dados Prospectivo e Multicêntrico de Pacientes com Infarto Agudo do Miocárdio: RIAM

**DOI:** 10.36660/abc.20190036

**Published:** 2020-04-06

**Authors:** Jacqueline Vaz, Anibal Pereira Abelin, Marcia Moura Schmidt, Pedro Piccaro de Oliveira, Carlos A. M. Gottschall, Clarissa Garcia Rodrigues, Alexandre Schaan de Quadros

**Affiliations:** 1 Instituto de Cardiologia Porto AlegreRS Brasil Instituto de Cardiologia, Porto Alegre, RS – Brasil; 2 Universidade Federal de Santa Maria Santa MariaRS Brasil Universidade Federal de Santa Maria (UFSM), Santa Maria, RS – Brasil; 3 Universidade Franciscana Santa MariaRS Brasil Universidade Franciscana (UNIFRA), Santa Maria, RS – Brasil

**Keywords:** Isquemia Miocárdica/fisiopatologia, Doenças Cardiovasculares/mortalidade, Infarto do Miocárdio/fisiopatologia, Estudo Multicêntrico, Base de Dados, Políticas Públicas de Saúde

## Abstract

**Fundamento:**

Registros multicêntricos representativos do mundo real podem fornecer informações importantes, mas existem poucos estudos descrevendo como implementar estas ferramentas.

**Objetivo:**

Descrever o processo de implementação de um banco de dados em infarto agudo do miocárdio com supradesnivelamento do segmento ST (IAMCST) em um hospital de referência e sua aplicação para outros centros com uma plataforma *online* .

**Métodos:**

Nossa instituição implementou em 2009 um Registro de Infarto Agudo do Miocárdio (RIAM), com a inclusão prospectiva e consecutiva de todos os pacientes com diagnóstico de IAMCST que internaram na instituição. No período de março de 2014 a abril de 2016 foi realizada a migração para o sistema *online* com o *software* REDCap e expansão do registro para outros centros. A plataforma REDCap é um *software* de uso gratuito disponibilizado pela Universidade *Vanderbilt* a instituições interessadas em pesquisa, mediante cadastramento prévio.

**Resultados:**

Foram realizadas as seguintes etapas do aprimoramento e expansão do registro: 1. Padronização das variáveis; 2. Implementação do *software* REDCap ( *Research Electronic Data Capture* ) institucional; 3. Desenvolvimento de formulários de coleta de dados ( *Case Report Form* - CRF); 4. Expansão do registro para outros centros de referência utilizando o *software* REDCap; 5. Treinamento da equipe e dos centros participantes pelo POP (Procedimento Operacional Padrão).

**Conclusões:**

A descrição da metodologia utilizada para implementar e expandir o RIAM pode auxiliar outros centros e pesquisadores a realizar estudos semelhantes, compartilhar informações entre instituições, o desenvolvimento de novas tecnologias em saúde e auxiliar nas políticas públicas em doenças cardiovasculares. (Arq Bras Cardiol. 2020; 114(3):446-455)

## Introdução

A cardiopatia isquêmica (CI) é uma das principais causas de morte no mundo.^1^No Brasil, segundo o DATASUS, o infarto agudo do miocárdio (IAM) é a principal causa de morte por doença cardíaca, porém são pouco conhecidas as características clínicas e o tratamento recebido pela maioria dos pacientes com IAM no país.^2^ Diversos registros internacionais de síndromes coronarianas agudas foram publicados, inclusive com a participação de alguns centros brasileiros,^3 , 4^ entretanto poucos estudos de abrangência nacional relatando os resultados do tratamento do IAM foram descritos até o momento.^5 , 6^

Os dados de registros necessitam de suporte tecnológico para armazenamento em bancos de dados informatizados, com *softwares* que forneçam fácil acesso aos dados, com segurança e confiabilidade. O REDCap ( *Research Eletronic Data Capture* ) é um *software* para coleta e armazenamento de dados clínicos amplamente utilizado para pesquisa clínica por se tratar de um aplicativo *web* rápido e seguro, atualmente utilizado por 3.175 instituições em 128 países.^7^ Poucos estudos relataram de maneira detalhada a metodologia de registros clínicos em cardiologia, e referências descrevendo as etapas para a implantação de um registro clínico e a utilização do *software* REDCap, como plataforma *online* , são escassas neste cenário.^8 - 11^

O Instituto de Cardiologia do Rio Grande do Sul/Fundação Universitária de Cardiologia (IC/FUC) iniciou em 2009 o Registro de Infarto Agudo do Miocárdio (RIAM), com coleta de dados consecutiva, prospectiva e ininterrupta desde sua implantação.^12^ Um registro nacional de IAM com supradesnivelamento do segmento ST (IAMCST) a partir da expansão de um registro como o RIAM poderia fornecer dados representativos desta patologia no Brasil. O objetivo deste estudo é descrever o processo de implementação de um banco de dados em IAMCST em um hospital de referência e sua aplicação para outros centros dentro do território nacional, utilizando uma plataforma *online* .

## Métodos

Descrição das etapas para a migração do banco de dados RIAM do *software* ACCESS (Microsoft) para o sistema *online* e expansão do registro para hospitais de referência para tratamento de IAMCST no território nacional, no período de março de 2014 a abril de 2016, através da padronização das variáveis, implementação de *software* dedicado (REDCap), desenvolvimento de formulários de coleta de dados e inclusão de novos centros com treinamento das equipes.

### O registro RIAM e a expansão para outros centros

O RIAM é um registro clínico prospectivo e consecutivo de pacientes com IAMCST atendidos no IC/FUC, em Porto Alegre/RS. Este registro foi iniciado em 2009 e já incluiu mais de 3.500 pacientes até esta data, sendo que estudos provenientes desta iniciativa têm embasado novas ideias de pesquisa científica e tecnológica na instituição.^12^ A expansão para outros centros será coordenada pelo IC/FUC, com participação adicional de 7 centros nacionais inicialmente.

### Elegibilidade e fluxo de trabalho

Os critérios de inclusão dos pacientes são: idade acima de 18 anos e IAMCST com menos de 12 horas de evolução. Pacientes com mais de 12 horas de evolução e dor torácica no momento da internação também são incluídos. O registro foi aprovado pelo Comitê de Ética em Pesquisa (CEP) do Instituto de Cardiologia/Fundação Universitária de Cardiologia, número 5025/14, com registro na Plataforma Brasil (CAAE: 38352714.0.0000.5333) e cada centro participante também o submeterá para aprovação em seus CEPs institucionais locais. Todos os pacientes assinarão um termo de consentimento informado e o registro será conduzido de acordo com os princípios da atual revisão da Declaração de *Helsinque* e com a última versão das Diretrizes de Boas Práticas Clínicas (ICH-GCP), bem como a Resolução 466/12 do Conselho Nacional de Saúde.^13 - 15^ A expansão do estudo foi realizada de acordo com os requisitos legais locais e regulatórios exigidos no Brasil.

## Resultados

### Desenho do registro

**Figura 1:**
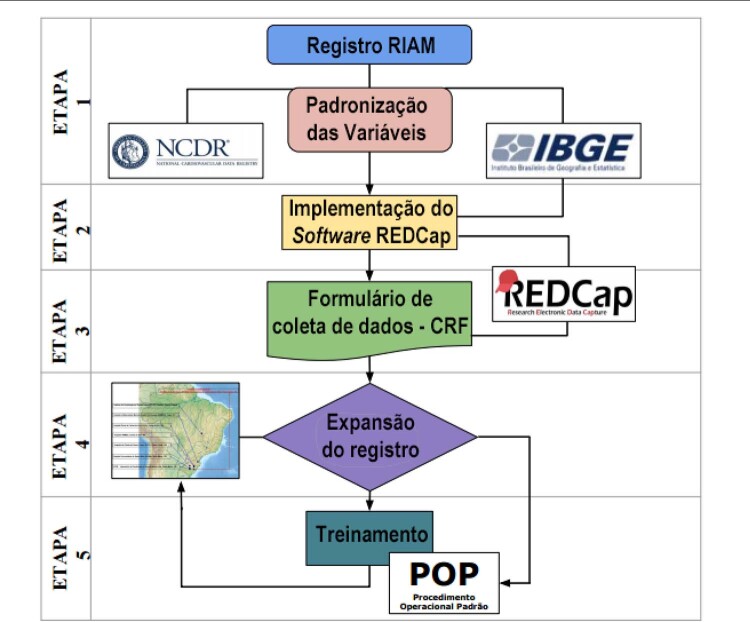
– Fluxograma de aprimoramento e expansão do Registro de Infarto Agudo do Miocárdio Multicêntrico. Fonte: Lucidchart. Disponível em: https://www.lucidchart.com

Foram realizadas as seguintes etapas da migração para o banco de dados *online* e expansão do registro, conforme demonstrado na Figura 1 : 1) Padronização das variáveis; 2) Implementação do *software* REDCap institucional; 3) Desenvolvimento de formulários de coleta de dados ( *Case Report Form* - CRF); 4) Expansão do registro para outros centros de referência utilizando o *software* REDCap; e 5) Elaboração de POPs (Procedimento Operacional Padrão) para treinamento das equipes e dos centros participantes.

### Padronização de variáveis

As variáveis já utilizadas no banco de dados baseado no *software Microsoft* ACCESS *™* foram comparadas com as variáveis padronizadas internacionalmente, com a checagem das nomenclaturas utilizadas, a fim de garantir que as informações do registro serão compatíveis com outros bancos de dados nacionais e internacionais.

As variáveis foram padronizadas de acordo com os padrões de dados clínicos de síndromes coronarianas agudas e doença arterial coronariana da *American College of Cardiology Foundation* (ACCF) e a *American Heart Association* (AHA), publicados em 2013, além dos formulários de elementos de dados do *National Cardiovascular Data Registry (NCDR)* , baseadas no ACTION *Registry®–* GWTG *™* (NCDR® ACTION Registry®-GWTG™ v2.4 Coder's Data Dictionary, substituído desde junho de 2018 pelo NCDR® Chest Pain - MI Registry™ v3.0 Coder's Data Dictionary), programa de qualidade de atendimento ao paciente com IAM coordenado pela ACCF.^16 , 17^ Para dados nacionais de etnia foi utilizada a classificação recomendada pelo Instituto Brasileiro de Geografia e Estatística (IBGE).^18^ Adicionalmente, foram revisados os dados padronizados utilizados pela Sociedade Brasileira de Cardiologia, visando facilitar a interoperabilidade internacional e nacional.^9^ A Tabela 1 demonstra algumas das variáveis selecionadas para o Registro conforme o NCDR ACTION *Registry®* .^17^

**Tabela 1:** – Planilha de variáveis pré-selecionadas do *ACTION Registry®–GWTG*

A. Dados Demográficos	Variável em Inglês	Legenda	Seleção
Sobrenome	last_name	Indica o sobrenome do paciente.	
Nome	first_name	Indica o primeiro nome do paciente.	
ID do paciente	patient_ID	Indica o número inserido automaticamente pelo *software* que identifica exclusivamente esse paciente.	
Data de nascimento	birth_date	Indica a data de nascimento do paciente.	
Sexo	Sex	Indica o sexo do paciente ao nascer.	Masculino; Feminino
**B. Dados de Admissão**	**Variável em Inglês**	**Legenda**	**Seleção**
CEP do paciente	patient_zip_code	Indica o código postal do paciente de sua residência principal.	
Data de admissão	admission_date	Indica a data em que o paciente foi admitido/internado em sua instituição para o atual episódio de atendimento.	
Seguro de Saúde Privado	insurance_payor_private	Indica se o seguro pagador do paciente inclui plano de saúde privado.	Não; Sim
**C. Dados Clinicos**	**Variável em Inglês**	**Legenda**	**Seleção**
Data de início do sintoma	symptom_onset_date	Indica a data em que o paciente relatou pela primeira vez os sintomas isquêmicos com duração maior ou igual a 10 minutos.	
Data do primeiro ECG	first_ECG_date	Indica a data do primeiro eletrocardiograma de 12 derivações (ECG)	
Insuficiência cardíaca	heart_failure	Indica se existe insuficiência cardíaca no primeiro contato médico	Não, Sim
Choque cardiogênico	cardiogenic_shock	Indica se o paciente estava em estado de choque cardiogênico no primeiro contato médico	Não, Sim
Frequência cardíaca	heart_rate	Indica o primeiro registro da frequência cardíaca (em batimentos por minuto)	
Pressão arterial sistólica	systolic_blood_pressure	Indica o primeiro registro da aferição da pressão arterial sistólica em milimitros de mercúrio (mmHg)	
Parada cardíaca	cardiac_arrest	Indica se o paciente estava em parada cardíaca no primeiro contato médico	Não, Sim

ID: identificação; ECG: eletrocardiograma; mmHg: milímetros de mercúrio. Fonte: ACTION Registry®–GWTG™. Previamente disponível em: www.ncdr.com/webncdr/action/home/datacollection (substituído a partir de junho de 2018 pelo NCDR® Chest Pain - MI Registry™, disponível em: https://cvquality.acc.org/NCDR-Home/registries/hospital-registries/chest-pain-mi-registry )

Nas variáveis selecionadas, identificou-se que os bancos RIAM e o *ACTION Registry®–* GWTG *™* apresentavam um perfil semelhante de variáveis, sugerindo que o RIAM ( Tabela 2 ) já apresentava um padrão comparável aos principais registros de IAM do mundo atualmente ( Tabela 3 ).

**Tabela 2:** – Variáveis RIAM - ACCESS

Demográficas	Banco de dados
ID Paciente	RIAM of ACCESS
Data de nascimento	RIAM of ACCESS
Sexo	RIAM of ACCESS
Raça ou cor	RIAM of ACCESS
Admissão	Banco de dados
IAM prévio	RIAM of ACCESS
Angina prévia	RIAM of ACCESS
Pressão arterial sistólica	RIAM of ACCESS
Pressão arterial diastólica	RIAM of ACCESS
Fatores de risco	Banco de dados
Diabete mellitus	RIAM of ACCESS
Dislipidemia	RIAM of ACCESS
AVC prévio	RIAM of ACCESS
CRM prévio	RIAM of ACCESS
Hipertensão	RIAM of ACCESS
Tabagismo	RIAM of ACCESS

Fonte: Quadro 2 - Registro RIAM Institucional, sistema Microsoft ACCESS™; Quadro 3 - ACTION Registry®–GWTG™. Previamente disponível em: www.ncdr.com/webncdr/action/home/datacollection (substituído a partir de junho de 2018 pelo NCDR® Chest Pain - MI Registry™, disponível em: https://cvquality.acc.org/NCDR-Home/registries/hospital-registries/chest-pain-mi-registry). ACCESS: Sistema de gerenciamento de banco de dados da Microsoft; ACTION Registry: Banco de dados da American College of Cardiology Foundation de pacientes com IAM; AVC: acidente vascular cerebral; CABG: coronary artery bypass graft; CRM: cirurgia de revascularização do miocárdio; IAM: infarto agudo do miocárdio; ID: identificação; RIAM: Registro de Infarto Agudo do Miocárdio.

**Tabela 3:** – Variáveis ***ACTION Registry***

Demográficas	Banco de dados
ID Paciente	ACTION Registry®
Data de nascimento	ACTION Registry®
Sexo	ACTION Registry®
Raça ou cor	ACTION Registry®
Admissão	Banco de dados
IAM prévio	ACTION Registry®
Angina prévia	ACTION Registry®
Pressão arterial sistólica	ACTION Registry®
Pressão arterial diastólica	ACTION Registry®
Fatores de risco	Banco de dados
Diabete mellitus	ACTION Registry®
Dislipidemia	ACTION Registry®
AVC prévio	ACTION Registry®
CRM prévio	ACTION Registry®
Hipertensão	ACTION Registry®
Tabagismo	ACTION Registry®

Fonte: Quadro 2 - Registro RIAM Institucional, sistema Microsoft ACCESS™; Quadro 3 - ACTION Registry®–GWTG™. Previamente disponível em: www.ncdr.com/webncdr/action/home/datacollection (substituído a partir de junho de 2018 pelo NCDR® Chest Pain - MI Registry™, disponível em: https://cvquality.acc.org/NCDR-Home/registries/hospital-registries/chest-pain-mi-registry). ACCESS: Sistema de gerenciamento de banco de dados da Microsoft; ACTION Registry: Banco de dados da American College of Cardiology Foundation de pacientes com IAM; AVC: acidente vascular cerebral; CABG: coronary artery bypass graft; CRM: cirurgia de revascularização do miocárdio; IAM: infarto agudo do miocárdio; ID: identificação; RIAM: Registro de Infarto Agudo do Miocárdio.

Posteriormente outra planilha foi gerada contendo as sessões do registro com o número de campos para inclusão no CRF do REDCap ( Tabela 4 ).

**Tabela 4:** – Sessão das variáveis padronizadas

Nome do Instrumento	Campos	Sessão dos Registros
Dados Demográficos	6	Identificação do paciente; data de nascimento; idade; convênio de saúde; anos de estudo; raça; sexo.
Contatos	4	Telefone principal; telefone secundário; telefone do familiar; e-mail do paciente.
Dados Clínicos 24h	18	Sintomas e atendimento inicial; Início do desconforto isquêmico; procedência; Dados do ECG; Parede do IAM; Sinais Vitais e Exame Físico; Estratégia de Reperfusão.
Medicamentos 24h	23	Medicações administradas nas 24h.
História Clínica Pregressa	22	Altura; Peso; IMC; DM; Tabagista; HAS; Dislipidemia; Angina; IAM; ACTP; CRM; Insuficiência Cardíaca; História familiar; AVC; Insuficiência Renal Crônica; câncer; anti-depressivo; Doença arterial periférica; FA e Flutter; Dispositivo cardíaco prévio.
Cateterismo e Intervenção	34	Dados do Cateterismo Cardíaco e ACTP; Achados na angiografia; Dados da angioplastia; Aspectos angiográficos;
Dados laboratoriais – internação	20	Coleta de exames laboratoriais na internação; Marcadores de lesão miocárdica positivos nas primeiras 24 horas.
Procedimentos e intercorrências - internação	26	Tipo de Infarto; Procedimentos até a alta; Intercorrências até a alta.
Dados da alta hospitalar	9	Óbito antes da alta hospitalar; Data da alta hospitalar; Tempo de internação; Medicações prescritas na alta hospitalar; MACE durante internação.
Desfechos e seguimento	24	Informações do Prontuário/Paciente/Familiar; Data do contato; óbito; causa do óbito; Internação hospitalar desde o último contato; IAM desde o último contato; Angina; PCR desde o último contato; AVC desde o último contato: ICP desde o último contato; CRM desde o último contato; Reestenose intra-stent; MACE; revisar as informações de contato.

ACTP: Angioplastia Coronariana Transluminal Percutânea; AVC: Acidente Vascular Cerebral; CRM: Cirurgia de Revascularização do Miocárdio; DM: Diabete Mellitus; ECG: Eletrocardiograma; FA: Fibrilação Atrial; HAS: Hipertensão Arterial Sistêmica; IAM; Infarto Agudo do Miocárdio; ICP: Intervenção Coronária Percutânea; IMC: Índice de Massa Corporal; MACE: Major Adverse Cardiac Events – Eventos Cardiovasculares Maiores; PCR: Parada Cardiorrespiratória.

Para cada variável foi incluído um *codebook* na língua inglesa, facilitando a integração com outros bancos de dados nacionais e internacionais, e acrescentada uma interface em português para a coleta de dados no Brasil.

### Implementação do *software* REDCap

O *software* utilizado como plataforma *online* foi o REDCap, o qual é internacionalmente reconhecido por sua segurança e aplicabilidade para coleta e armazenamento de dados clínicos. O sistema segue o modelo internacional do *Duke Clinical Research Institute* , obedecendo às exigências de segurança internacionais e da Agência Nacional de Vigilância Sanitária (ANVISA).^19 , 20^

Entre outras características, o REDCap fornece (1) uma interface intuitiva para a entrada de dados validados, com verificação automatizada dos tipos de dados e de verificações de intervalo; (2) pistas de auditoria para o acompanhamento dos procedimentos de manipulação e exportação de dados; (3) procedimentos automatizados de exportação de dados para pacotes estatísticos comuns e (4) procedimentos para importação de dados de fontes externas.^21^ A coleta de dados é realizada em qualquer dispositivo com acesso à *internet* como computador, *tablet* ou *smartphone* , ou mesmo *off-line* com instalação do aplicativo REDCap, com recurso de sincronização assim que obtido acesso à *internet* .^22^

O REDCap é uma solução para a gestão de banco de dados, sendo utilizada para a coleta e gerenciamento de dados em pesquisa, de maneira segura e *online* . Foi desenvolvido em torno de diretrizes da *Health Insurance Portability and Accountability Act (HIPAA)* .^23^ Seu uso é gratuito e oferecido pelo *Center for Research Informatics* (CRI), que após a licença pela *Vanderbilt Universit* foi hospedado em um servidor local e protegido pelo *firewall* do sistema do IC/FUC.

Para acesso são necessários um usuário (u *sername* ) e senha ( *password* ) individuais, solicitados e aprovados pelo gestor local do *software* na instituição. Dentro do REDCap foi possível a criação do CRF contendo as variáveis padronizadas do estudo.

### Desenvolvimento do formulário de coleta de dados ( *Case report form* - CRF)

**Figura 2:**
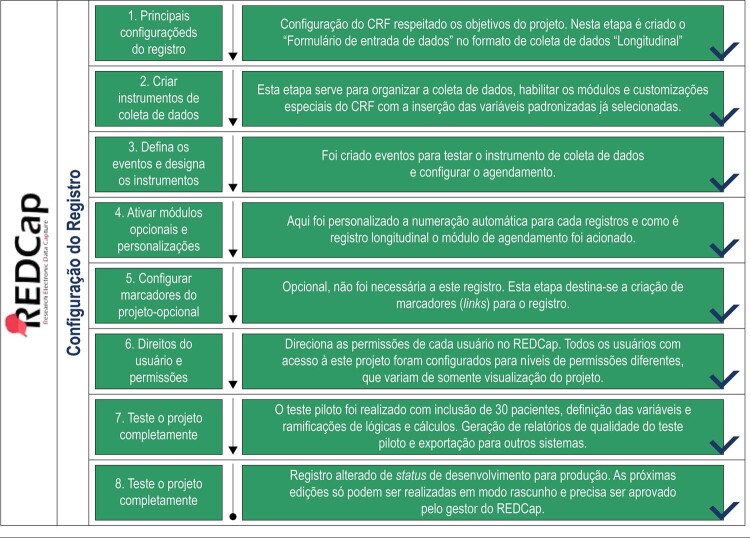
– Diagrama de opções de criação do Case Report Form. Fonte: REDCap IC/FUC http://redcap.cardiologia.org.br/redcap/redcap_v6.1.0/ProjectSetup/index.php?pid=23

Os formulários eletrônicos – *Case Report Form* (CRF) foram desenvolvidos através do REDCap. A Figura 2 mostra as etapas necessárias para a configuração do CRF.

As etapas para a criação do CRF seguiram as orientações do *software* , onde dentro da terceira etapa foi realizado o teste piloto com pacientes escolhidos aleatoriamente do banco RIAM do sistema Microsoft ACCESS *™* com o intuito apenas de validação do CRF. Os procedimentos automatizados de exportação para *downloads* de dados para programas como o *Microsoft Excel* e pacotes estatísticos comuns como o SPSS, SAS e o R, foram realizados a fim de garantir a segurança e confiabilidade do *software* .

### Expansão do Registro para outros centros de referência utilizando o software REDCap institucional

Os centros participantes foram selecionados após contato pessoal do coordenador do estudo (AQ) com cardiologistas intervencionistas que realizaram formação no IC/FUC e que trabalhavam em hospitais com serviço de cardiologia intervencionista com atendimento de IAMCST 24 horas por dia, 7 dias por semana. Primeiramente foi realizado uma reunião apresentando a proposta da expansão aos coordenadores locais dos demais centros (denominados de *Principal Investigator* - PI) que aceitaram participar do RIAM. Após, os centros convidados foram sendo informados dos processos de participação via *e-mail* .

**Figura 3:**
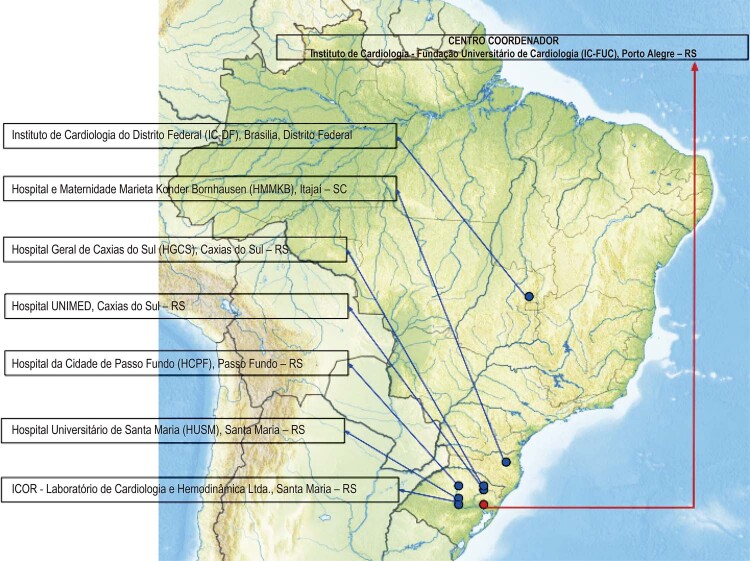
– Distribuição Nacional - Centros do Registro de Infarto Agudo do Miocárdio Multicêntrico. Via: Google Drawings - https://docs.google.com/drawings

As instituições convidadas que aceitaram participar da fase multicêntrica do Registro RIAM, sob a coordenação do IC/FUC, estão distribuídas principalmente na região Sul do Brasil ( Figura 3 ):

1. Instituto de Cardiologia do Distrito Federal (IC-DF), Brasília, Distrito Federal, Brasil.
2. Hospital e Maternidade Marieta Konder Bornhausen (HMMKB), Itajaí, Santa Catarina, Brasil;
3. Hospital Geral de Caxias do Sul (HGCS), Caxias do Sul, Rio Grande do Sul, Brasil;
4. Hospital UNIMED, Caxias do Sul, Rio Grande do Sul, Brasil;
5. Hospital da Cidade de Passo Fundo (HCPF), Passo Fundo, Rio Grande do Sul, Brasil;
6. Hospital Universitário de Santa Maria (HUSM), Santa Maria, Rio Grande do Sul, Brasil;
7. ICOR - Instituto do Coração de Santa Maria (ICOR), Santa Maria, Rio Grande do Sul, Brasil;

Para iniciar a expansão multicêntrica foram criados protocolos do registro RIAM para inclusão dos centros participantes. Estes protocolos foram enviados por *e-mail* em formato PDF ( *Portable Document Format* ) para posterior impressão, preenchimento e assinatura. Os protocolos, após assinados, eram reenviados ao centro coordenador via *scanner, e-mail* , correios ou entregues pessoalmente aos coordenadores do registro RIAM.

### Treinamento – POP (Procedimento Operacional Padrão)

Antes de iniciar a coleta de dados, o investigador principal e seus pesquisadores receberam um *e-mail* com o *link* do REDCap para acesso, assim como o u *sername* e *password* individual, que após o recebimento poderá solicitar novo *password* , garantindo a confidencialidade do pesquisador dentro do REDCap institucional.

**Figura 4:**
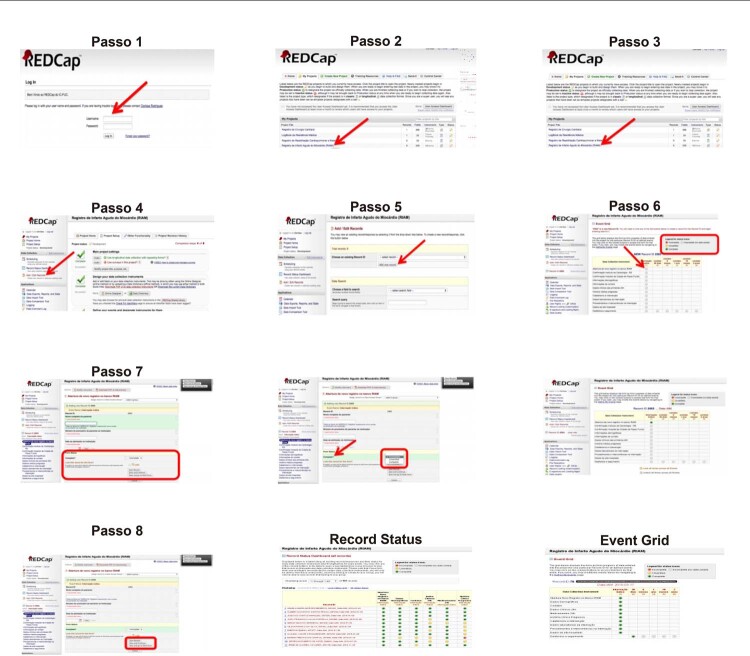
– Procedimento Operacional Padrão para inserção de dados no REDCap. Fonte: REDCap IC/FUC - http://redcap.cardiologia.org.br

O treinamento foi focado nos objetivos do registro, esclarecendo o processo de coleta e inserção de dados no REDCap. O POP para coleta de dados assegura a coleta padronizada e consistente, e também contém uma descrição de todos os elementos de dados, incluindo suas definições e procedimentos a serem usados durante a inserção dos dados ( Figura 4 ). Além disso, foi disponibilizado aos pesquisadores treinamento *online* e presencial para esclarecer possíveis questões sobre o processo de coleta de dados. As atividades de entrada de dados foram monitoradas de maneira *online* .

### Relatórios de qualidade de dados pelo *software* REDCap

**Figura 5:**
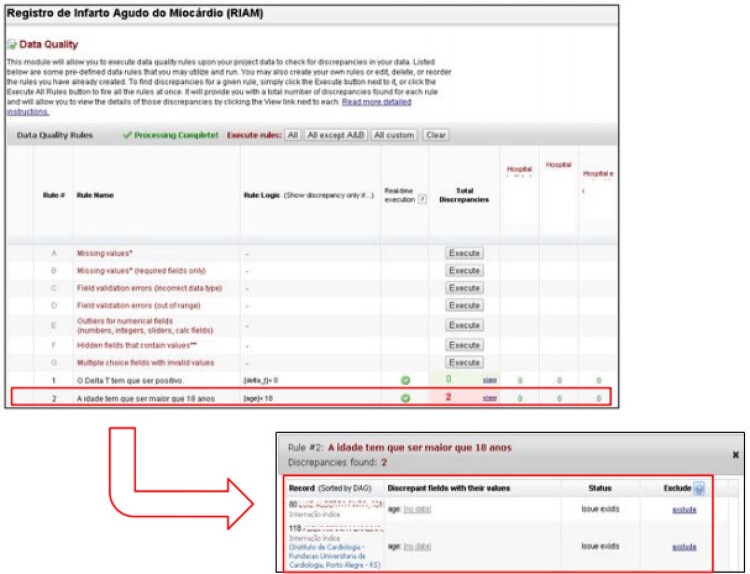
– Relatório de qualidade de dados. Fonte: REDCap IC/FUC - http://redcap.cardiologia.org.br

Para geração de relatórios de dados automatizados para controle de qualidade e com o objetivo de prevenir dados incompletos, as variáveis principais foram incluídas como dados obrigatórios, assim como a definição de limites - *ranges* - como intervalos mínimos e máximos para variáveis numéricas. Os relatórios de dados faltantes – *missing -* são gerados esporadicamente para conferência interna do preenchimento das variáveis – *records –* obrigatórias. Relatórios de validação de campo para conferência de dados incorretos também foram gerados, assim como relatórios de campos numéricos para conferência de variáveis fora do padrão, inválidas ou não preenchidas ( Figuras 5 ).

## Discussão

Neste estudo descrevemos o processo de implantação de um banco de dados em IAMCST em um hospital de referência e sua aplicação para outros centros dentro do território nacional, utilizando uma plataforma *online* . Foram detalhados os processos de padronização das variáveis, implementação do *software* REDCap institucional, desenvolvimento de formulários de coleta de dados ( *Case Report Form* - CRF), expansão do registro para outros centros de referência utilizando o *software* REDCap e treinamento da equipe e dos centros participantes pelo POP (Procedimento Operacional Padrão).

Os ensaios clínicos randomizados (ECR) são o padrão-ouro para demonstrar a eficácia de uma determinada intervenção e constituem a base teórica para a formulação de diretrizes. Dados observacionais como os obtidos em registros clínicos complementam a evidência científica dos ECRs ao demonstrar efetividade na prática clínica.^24^ Registros nacionais representativos da população de pacientes com IAMCST, com análise das características clínicas, terapêuticas e dos desfechos, são necessários para avaliar a prática clínica no nosso meio, além de permitir mensurar a adesão às diretrizes, desenvolver ferramentas para a estratificação de risco e guiar políticas públicas para melhorar o tratamento desta patologia no nosso meio.^4 , 25 , 26^ Para a avaliação dos desfechos é necessária a padronização das variáveis utilizando como referência a terminologia padrão, permitindo a comparação com os resultados de outros estudos como registros internacionais e ECRs, além de proporcionar colaboração com troca de informações entre diferentes centros de atendimento de pacientes com IAMCST. Durante o processo de aprimoramento e padronização das variáveis do nosso registro foi utilizado como referência o registro de IAMCST da NCDR, coordenado pela ACCF, sendo identificado o mesmo perfil de variáveis entre os bancos de dados RIAM e NCDR.^16 , 17^

Os registros que buscam uma representatividade e abrangência nacional devem incluir o maior número consecutivo de pacientes, sendo importante a coleta de dados eficiente e de qualidade associada à mínima interferência na prática clínica.^11^ O *software* REDCap, desenvolvido pela *Vanderbilt University* , possui as características necessárias para servir de ferramenta para a coleta e armazenamento dos dados. Entre as funcionalidades do *software* estão uma interface intuitiva para a edição de formulários de coleta de dados (CRF), fácil inserção de dados com a opção de dupla digitação, validação em tempo real dos dados, possibilidade de auditoria dos dados, segurança no armazenamento e troca de informações, assim como função de exportação para pacotes estatísticos.^21^

A decisão de focar este artigo na metodologia da implantação de um banco de dados com o uso do *software* REDCap visa servir de modelo para o desenvolvimento de registros clínicos de qualidade, além de facilitar a integração dos centros de pesquisa do registro RIAM.

### Limitações

A implementação e expansão desse estudo observacional do tipo registro apresenta como uma das suas limitações a falta de integração do prontuário eletrônico com o banco de dados, ocasionando aumento da carga de trabalho e, eventualmente, a necessidade de pessoal dedicado à pesquisa durante a assistência dos pacientes. A avaliação de dados de registros clínicos deve levar em conta a necessidade de consentimento informado para a obtenção de dados, acarretando no risco da não inclusão da totalidade de pacientes elegíveis no caso de negativa na participação, além da possibilidade do paciente alterar seus hábitos pela ciência de estar participando de um estudo, mesmo que observacional (efeito *Hawthorne* ).^27^

Dentre as limitações, deve ser destacado que, apesar do RIAM apresentar a possibilidade de ter abrangência nacional, o mesmo incluiu apenas centros da região Sul do Brasil, com exceção de um centro no Distrito Federal, limitando a representatividade desta base de dados de pacientes com IAMCST. A inclusão de novos centros, obedecendo o critério de apresentarem tratamento intervencionista do IAMCST nas 24 horas do dia nos 7 dias da semana, é o próximo passo após a consolidação da fase operacional inicial do RIAM.

## Conclusão

Neste estudo descrevemos a logística e sistemática do desenvolvimento de um registro clínico de pacientes com IAMCST na plataforma digital REDCap, adaptado de um registro clínico já existente. Estes dados podem ser úteis para instituições que planejam criar novos registros ou aprimorar os que já existem. A padronização da operação dos registros e uso de bancos de dados dedicados possibilitam a otimização desta ferramenta, tanto em relação a sua qualidade quanto a rapidez da sua implementação. A utilização de sistemas semelhantes também pode facilitar o compartilhamento de informações entre instituições, o desenvolvimento de novas tecnologias em saúde e auxiliar na tomada de decisão de políticas públicas em doenças cardiovasculares.
