# Supplementary material for: Criação e Implementação de um Banco de Dados Prospectivo e Multicêntrico de Pacientes com Infarto Agudo do Miocárdio: RIAM
Source: Arq Bras Cardiol. 2020 Apr 6;114(3):446–55. [Article in Portuguese] doi: 10.36660/abc.20190036 (PMC7792739; doi:10.36660/abc.20190036)
Supplement: Translation

# Translation: Creation and Implementation of a Prospective and Multicentric Database of Patients with Acute Myocardial Infarction: RIAM

**Authors:** Jacqueline Vaz, Anibal Pereira Abelin, Marcia Moura Schmidt, Pedro Piccaro de Oliveira, Carlos A. M. Gottschall, Clarissa Garcia Rodrigues, Alexandre Schaan de Quadros

**Affiliations:** 1 Brazil Instituto de Cardiologia, Porto Alegre, RS – Brazil; 2 Brazil Universidade Federal de Santa Maria (UFSM), Santa Maria, RS – Brazil; 3 Brazil Universidade Franciscana (UNIFRA), Santa Maria, RS – Brazil

**Keywords:** Myocardial Ischemia/physiopathology, Cardiovascular Diseases/mortality, Myocardial Infarction/physiopathology, Multicenter Study, Database, Public Health Policy

## Abstract

**Background:**

Multicenter registries representing the real world can be a significant source of information, but few studies exist describing the methodology to implement these tools.

**Objective:**

To describe the process of implementing a database of ST-segment elevation acute myocardial infarction (STEMI) at a reference hospital, and the application of this process to other centers by means of an online platform.

**Methods:**

In 2009, our institution implemented an Registry of Acute Myocardial Infarction (RIAM), with the prospective and consecutive inclusion of every patient admitted to the institution who received a diagnosis of STEMI. From March 2014 to April 2016, the registries were uploaded to a web-based system using the REDCap software and the registry was expanded to other centers. Upon subscription, the REDCap platform is a noncommercial software made available by Vanderbilt University to institutions interested in research.

**Results:**

The following steps were taken to improve and expand the registry: 1. Standardization of variables; 2. Implementation of institutional REDCap (Research Electronic Data Capture); 3. Development of data collection forms (Case Report Form - CRF); 4. Expansion of registry to other reference centers using the REDCap software; 5. Training of teams and participating centers following an SOP (Standard Operating Procedure).

**Conclusion:**

The description of the methodology used to implement and expand the RIAM may help other centers and researchers to conduct similar studies, share information between institutions, develop new health technologies, and assist public policies regarding cardiovascular diseases. (Arq Bras Cardiol. 2020; 114(3):446-455)

## Introduction

Ischemic cardiomyopathy (IC) is one of the leading causes of death in the world.^1^According to DATASUS (Brazilian Basic Health Indicators and Data), acute myocardial infarction (AMI) is the leading cause of death from heart disease in Brazil, but information on clinical characteristics and treatment received by most patients with AMI in the country are poorly known.^2^Many international registries of acute coronary syndromes have been published, which include the collaboration of some Brazilian centers:^3 , 4^however, only a few nationwide studies reporting AMI treatment outcomes have been published so far.^5 , 6^

The management of registry data demands technological support for their storage in computerized databases, with software that provides safe, reliable and easy access to data. Research Electronic Data Capture (REDCap) is a software for clinical data capture and storage which is widely used for clinical research. It is a fast and secure web application currently used by 3,175 institutions in 128 countries.^7^Few studies have reported in detail the methodology of recording clinical data in cardiology, and references describing the steps for the implementation of a clinical registry and for the use of REDCap as an online platform are scarce.^8 - 11^

The Instituto de Cardiologia do Rio Grande do Sul/Fundação Universitária de Cardiologia (IC/FUC) started the Registry of Acute Myocardial Infarction (RIAM) in 2009, with consecutive, prospective and uninterrupted data collection since it was implemented.^12^A national ST-segment elevation myocardial infarction (STEMI) registry derived from the expansion of a registry such as RIAM could be a source of representative data for this pathology in Brazil. The aim of this study is to describe the implementation of a STEMI database in a reference hospital as well as the use of an online platform to apply it to other centers across the national territory.

## Methods

This section describes the steps taken to migrate the RIAM database from Microsoft Access into the online system and to expand the registry to reference hospitals treating STEMI across the national territory. This took place from March 2014 to April 2016 and included the standardization of variables; the implementation of dedicated software (REDCap); the development of data collection forms; and the inclusion of new centers with staff training.

### RIAM and the expansion to other centers

RIAM is a prospective and consecutive clinical registry of STEMI patients treated at IC/FUC, in Porto Alegre/RS, Brazil. The registry was started in 2009 and currently has more than 3,500 patients. This initiative contributed to new studies with ideas for scientific and technological research in the institution.^12^IC/FUC will coordinate the expansion to seven other national centers, initially.

### Eligibility and workflow

The inclusion criteria were patients aged at least 18 years old and STEMI with less than 12 hours of symptoms. Patients with more than 12 hours of symptoms and reporting chest pain at admission are also included. The registry was approved by the Ethics Committee of IC/FUC number 5025/14, with registration in Plataforma Brasil (CAAE: 38352714.0.0000.5333), and each participating center will also submit it for approval in their local institutional ethics committees. All patients are required to sign an informed consent form and their data will be collected in accordance with the principles of the current revision of the Declaration of Helsinki and the latest version of the Good Clinical Practice Guidelines (ICH-GCP), as well as Resolution 466/12 of the Brazilian National Health Council.^13 - 15^The study was expanded according to Brazilian legal and regulatory requirements.

## Results

### Registry design

The following steps were taken to migrate the system to the online database and expand the registry, as shown in Figure 1 : Step 1. Standardization of variables; Step 2. Implementation of institutional REDCap software *;* Step 3. Development of data collection forms (Case *Report Form* - CRF); Step 4. Expansion of registry to other reference centers using the REDCap software; Step 5. Creation of SOPs (Standard Operating Procedures) for training teams and participating centers.

**Figure 1:** – Improvement and expansion flowchart for the multicenter Registry of Acute Myocardial Infarction. Source: Lucidchart. Available in: https://www.lucidchart.com

### Standardization of variables

The nomenclature assigned to the variables already used in the Microsoft Access™ database were compared with internationally standardized variables to ensure the information in the registry is compatible with other national and international databases.

Variables were standardized based on the American College of Cardiology Foundation (ACCF) and American Heart Association (AHA) clinical data standards for acute coronary syndromes and coronary artery diseases, published in 2013. They were also based on data element forms from the National Cardiovascular Data Registry (NCDR), the ACTION Registry®-GWTG™ (NCDR® ACTION Registry®-GWTG™ v2.4 Coder's Data Dictionary, replaced by the NCDR® Chest Pain - MI Registry™ v3.0 Coder's Data Dictionary as of June 2018), an ACCF-coordinated quality care program for patients with MI.^16 , 17^For national data regarding ethnicity the classification recommended by the Brazilian Institute of Geography and Statistics (IBGE)^18^was used. In addition, a review of the standardized data used by the Brazilian Society of Cardiology was carried out to facilitate international and national interoperability.^9^ Table 1 shows some of the variables selected for the registry according to the NCDR ACTION Registry®.^17^

**Table 1:** – Pre-selected variables in ACTION Registry®–GWTG

A. Demographic Data	Variable in English	Legend	Selection
Last name	Last_name	Indicates the patient's last name	
First name	first_name	Indicates the patient's first name	
Patient’s identification number	Patient_ID	Indicates the number entered automatically by the software that uniquely identifies this patient	
Date of birth	Birth_date	Indicates the date of birth of the patient	
Sex	Sex	Indicates the patient's sex at birth	Male; Female
**B. Admission Data**	**Variable in English**	**Legend**	**Selection**
Patient’s ZIP code	patient_zip_code	Indicates the zip code of the patient’s primary home	
Date of admission	admission_date	Indicates the date the patient was admitted to the institution for the treatment of the current episode	
Private health plan	insurance_payor_private	Indicates whether the patient's insurance payor includes a private health plan	No; Yes
**C. Clinical Data**	**Variable in English**	**Legend**	**Selection**
Date of symptom onset	symptom_onset_date	Indicates the date the patient first reported ischemic symptoms for 10 minutes or more.	
Date of first ECG	first_ECG_date	Indicates date of first 12-lead electrocardiogram	
Heart failure	heart_failure	Indicates the existence of heart failure on first medical contact	No, Yes
Cardiogenic shock	cardiogenic_shock	Indicates whether the patient was in cardiogenic shock on first medical contact	No, Yes
Heart rate	heart_rate	Indicates the first heart rate record (in beats per minute)	
Systolic blood pressure	systolic_blood_pressure	Indicates first record of systolic blood pressure in mmHg	
Cardiac arrest	cardiac_arrest	Indicates whether the patient was in cardiac arrest on first medical contact	No, Yes

ID: Identification; ECG: Electrocardiogram; mmHg - Millimeters of mercury. Source: ACTION Registry®–GWTG™. Previously available at: www.ncdr.com/webncdr/action/home/datacollection (replaced by NCDR® Chest Pain - MI Registry™ as of June 2018, available at: https://cvquality.acc.org/NCDR-Home/registries/hospital-registries/chest-pain-mi-registry )

Among the selected variables, the RIAM and ACTION Registry® – GWTG™ databases were found to have a similar profile across variables, suggesting that the RIAM ( Table 2 ) already had a pattern comparable to the main MI registries in the world today ( Table 3 ).

**Table 2:** – Variables RIAM - ACCESS

Demographics	Database
Patient ID	RIAM of ACCESS
Birth Date	RIAM of ACCESS
Sex	RIAM of ACCESS
Race	RIAM of ACCESS
Admission	Database
Prior MI	RIAM of ACCESS
Prior angina	RIAM of ACCESS
Systemic Systolic Blood Pressure	RIAM of ACCESS
Systemic Diastolic Blood Pressure	RIAM of ACCESS
Risk factors	Database
Diabetes	RIAM of ACCESS
Dyslipidemia	RIAM of ACCESS
Prior CVA	RIAM of ACCESS
Prior CABG	RIAM of ACCESS
Hypertension	RIAM of ACCESS
Tobacco use	RIAM of ACCESS

Source: Table 2 - Institutional RIAM Registry, Microsoft ACCESS™; Table 3 - ACTION Registry®–GWTG™. Previously available at: www.ncdr.com/webncdr/action/home/datacollection (replaced by NCDR® Chest Pain - MI Registry™ as of June 2018, available at: https://cvquality.acc.org/NCDR-Home/registries/hospital-registries/chest-pain-mi-registry). Access: Database management system from Microsoft; ACTION Registry: MI patient database from the American College of Cardiology Foundation; CVA: Cerebrovascular Accident; CABG: Coronary Artery Bypass Graft; MRS: Myocardial revascularization surgery; Acute MI: Acute Myocardial Infarction; ID: Identification; MI: Myocardial Infarction; RIAM: Registry of Acute Myocardial Infarction.

**Table 3:** – Variables ACTION Registry

Demographics	Database
Patient ID	ACTION Registry®
Birth Date	ACTION Registry®
Sex	ACTION Registry®
Race	ACTION Registry®
Admission	Data Base
Prior MI	ACTION Registry®
Prior angina	ACTION Registry®
Systemic Systolic Blood Pressure	ACTION Registry®
Systemic Diastolic Blood Pressure	ACTION Registry®
Risk factors	Data Base
Diabetes	ACTION Registry®
Dyslipidemia	ACTION Registry®
Prior CVA	ACTION Registry®
Prior CABG	ACTION Registry®
Hypertension	ACTION Registry®
Tobacco use	ACTION Registry®

Source: Table 2 - Institutional RIAM Registry, Microsoft ACCESS™; Table 3 - ACTION Registry®–GWTG™. Previously available at: www.ncdr.com/webncdr/action/home/datacollection (replaced by NCDR® Chest Pain - MI Registry™ as of June 2018, available at: https://cvquality.acc.org/NCDR-Home/registries/hospital-registries/chest-pain-mi-registry). Access: Database management system from Microsoft; ACTION Registry: MI patient database from the American College of Cardiology Foundation; CVA: Cerebrovascular Accident; CABG: Coronary Artery Bypass Graft; MRS: Myocardial revascularization surgery; Acute MI: Acute Myocardial Infarction; ID: Identification; MI: Myocardial Infarction; RIAM: Registry of Acute Myocardial Infarction.

Subsequently another spreadsheet was generated containing the sessions of registry with the number of fields to be included in REDCap CRF ( Table 4 ).

**Table 4:** – Session of standardized variables

Name of Instrument	Fields	Session of Registries
Demographic data	6	Patient identification; date of birth; age; health insurance payor; education; race; sex.
Contacts	4	Main phone number; second phone number; family member’s phone number; patient’s e-mail address.
Clinical data 24h	18	Symptoms and initial care; Onset of ischemic discomfort; origin; ECG data; MI wall; Vital Signs and Physical Examination; Reperfusion Strategy.
Medication 24h	23	Medication given for 24h.
Clinical History	22	Height; Weight; BMI; DM; Tobacco user; HAS; Dyslipidemia; Angina; AMI; ACTP; CRM; Cardiac insufficiency; Family history; CVA; Chronic Kidney Failure; cancer; antidepressant; Peripheral arterial disease; FA and Flutter; Previous cardiac device.
Catheterization and Intervention	34	Cardiac Catheterization and ACTP Data; Angiography findings; Angioplasty data; Angiographic aspects;
Laboratory data - admission	20	Laboratory tests performed on admission; Positive myocardial injury markers within first the 24 hours.
Procedures and complications - hospitalization	26	Infarction Type; Procedures until discharge; Complications until discharge.
Data from hospital discharge form	9	Death before hospital discharge; Date of hospital discharge; Length of hospital stay; Medication prescribed at hospital discharge; MACE during hospitalization.
Outcome and follow-up	24	Records/Patient/Family information; Date of contact; death; Cause of death; Hospitalization since last contact; MI since last contact; Angina; CA since last contact; CVA since last contact: ICP since last contact; CRM since last contact; Intra-stent restenosis; MACE; Review contact information.

ACTP: percutaneous transluminal coronary angioplasty; CVA: cerebrovascular accident; MRS: myocardial revascularization surgery; DM: diabetes mellitus; ECG: electrocardiogram; AF: atrial fibrillation; SAH: systemic arterial hypertension; MI; acute myocardial infarction; PCI: percutaneous coronary intervention; BMI: body mass index; MACE: major adverse cardiac events; CA: cardiac arrest.

A codebook in English was included for each variable to enable smoother integration with other national and international databases, and an interface in portuguese was added for data collection in Brazil.

### Deployment of REDCap software

REDCap was the software used by means of an online platform. It is internationally acknowledged for its security and applicability for clinical data capture and storage. The system is based on the international model by the Duke Clinical Research Institute, complying with international security requirements as well as with the Brazilian National Health Surveillance Agency (ANVISA).^19 , 20^

Some of the features of REDCap are: (1) an intuitive interface for validated data entry, with automated data type and range checks; (2) audit trails for tracking data manipulation and export procedures, (3) automated data export procedures for common statistical packages and (4) procedures for importing data from external sources.^21^Data collection is performed on any device with internet access such as a computer, tablet or smartphone, or even offline via the REDCap application, which synchronizes the data once there is internet access.^22^

REDCap is a noncommercial secure web-based database management solution offered by the Center for Research Informatics (CRI). It is used for the collection and management of research data and was developed following guidelines from the Health Insurance Portability and Accountability Act (HIPAA).^23^After obtaining a license from Vanderbilt University, the software was hosted on a local server protected by the IC/FUC system firewall.

Access requires an individual username and password requested and approved by the local software manager at the institution. REDCap allowed the creation of a CRF, which contained the standardized variables of the study.

### Development of a data collection form (Case report form - CRF)

The electronic data form – *Case Report Form* (CRF) was developed using REDCap. Figure 2 shows the necessary steps to set up the CRF.

**Figure 2:** – Options Diagram for the creation of the Case Report Form. Source: REDCap IC/FUC http://redcap.cardiologia.org.br/redcap/redcap_v6.1.0/ProjectSetup/index.php?pid=23

The steps for the creation of a CRF followed the guidelines of the software. Within the third step, a pilot test was run with patients randomly chosen from the Microsoft Access™ RIAM database for the purpose of CRF validation. Automated export procedures for data download for programs such as Microsoft Excel and common statistical packages such as SPSS, SAS and R were performed to ensure software security and reliability.

### Registry Expansion to other reference centers using the institutional REDCap

Invited centers were selected because of the existence of interventional cardiology sectors with STEMI treatment 24 hours a day, 7 days a week. Initially, a meeting was held with local coordinators of other centers (called Principal Investigator - PI) to present the proposed expansion. Afterwards, the invited centers were informed of the participation processes via e-mail.

The institutions that agreed to participate in the multicenter phase of the RIAM Registry are located in many regions in Brazil ( Figure 4 ):

**Figure 4:** – Standard Operating Procedure for entering data in REDCap. Source: REDCap IC/FUC - http://redcap.cardiologia.org.br

1. Instituto de Cardiologia - Fundação Universitária de Cardiologia (IC/FUC), Porto Alegre, Rio Grande do Sul, Brazil – Coordinating Center;
2. Instituto de Cardiologia do Distrito Federal (IC-DF), Brasília, Distrito Federal, Brazil.
3. Hospital e Maternidade Marieta Konder Bornhausen (HMMKB), Itajaí, Santa Catarina, Brazil;
4. Hospital Geral de Caxias do Sul (HGCS), Caxias do Sul, Rio Grande do Sul, Brazil;
5. Hospital UNIMED, Caxias do Sul, Rio Grande do Sul, Brazil;
6. Hospital da Cidade de Passo Fundo (HCPF), Passo Fundo, Rio Grande do Sul, Brazil;
7. Hospital Universitário de Santa Maria (HUSM), Santa Maria, Rio Grande do Sul, Brazil;
8. ICOR - Instituto do Coração de Santa Maria (ICOR), Santa Maria, Rio Grande do Sul, Brazil;

RIAM registry protocols were created to include participating centers to then start multicenter expansion. These protocols were sent by email in PDF (Portable Document Format) for later printing, completion and signature. Once signed, the protocols were sent back to the coordinating center via scanner, email, mail or personally delivered to the RIAM registry coordinators.

### Training - SOP (Standard Operating Procedure)

Before data collection, the principal investigator and their researchers received an email with a link to access REDCap and an individual username and password, which upon receipt may request the creation of a new password, ensuring the confidentiality of the researcher in institutional REDCap.

The training focused on the goal of the registry, clarifying the process of collecting and entering data into REDCap. The SOP for data collection ensures standardized and consistent data collection and contains a description of all data elements, including their definitions and procedures to be used while entering data ( Figure 4 ). In addition, online and face-to-face training was provided to researchers to clarify possible questions about the data collection process. Data entry activities were monitored online.

### Data quality reports by REDCap software

For the generation of automated quality control reports and to prevent incomplete data, the main variables were included as required data, and the limits were defined as minimum and maximum ranges for numerical variables ( *ranges* ). Missing data reports ( *missing* ) were sporadically generated for internal checking of the required variable ( *records* ). Field validation reports for checking incorrect data were also generated, as well as numeric field reports for checking non-standard, invalid, or unfilled variables. ( Figure 5 ).

**Figure 5:** – Data Quality Report. Source: REDCap IC/FUC - http://redcap.cardiologia.org.br

## Discussion

In this study, we described the process of implementing a STEMI database in a reference hospital and its application to other centers across the national territory through the use of a web-based platform. We also detailed the processes for standardization of variables, implementation of institutional REDCap software, development of case report forms (CRF), expansion of the registry to other reference centers using the REDCap software, and training of staff and participating centers using an SOP (Standard Operating Procedure).

Randomized controlled trials (RCTs) are the gold standard for demonstrating the effectiveness of a given intervention and form the theoretical basis for formulating guidelines. Observational data such as those obtained from clinical records complement scientific evidence of RCTs by demonstrating effectiveness in clinical practice.^24^The assessment of clinical practice in Brazil requires access to national records representing the STEMI patient population to provide the analysis of clinical and therapeutic characteristics in addition to its outcomes. Besides that, it allows to measure compliance to guidelines, develop risk stratification tools and inform public policies to improve the treatment of this pathology in our country.^4 , 25 , 26^The evaluation of outcomes requires the standardization of variables using standard terminology, thus allowing comparison with results from other studies such as international registries and RCTs. It also promotes collaboration from information exchange across STEMI patient care centers. During the process of improvement and standardization of variables in our registry, the NCDR STEMI registry coordinated by the ACCF was used as a reference, and the same variable profile was found both in RIAM and NCDR databases.^16 , 17^

Any registry seeking national representativeness and coverage should include the largest number of consecutive patients and an association of quality and efficiency in data collection. In addition to that, it is important to keep minimal interference in clinical practice.^11^REDCap, developed by Vanderbilt University, has the necessary features to serve as a tool for data collection and storage. Software features include an intuitive interface for editing data collection forms (CRF), easy data entry with double-typing, real-time data validation, data auditability, security in storage and information exchange, and an export function for statistical packages.^21^

The decision to focus this article on the methodology of implementing a database using REDCap aims to serve as a benchmark in the development of quality clinical registries, as well as to make integration of RIAM research centers friendly.

### Limitations

One limitation in the implementation and expansion of this observational, registry-based study is the absence of integration between electronic medical records and database, which causes increased workload and, eventually, the need for dedicated research staff during patient care. The evaluation of clinical registry data should also consider the need for informed consent in data collection, which jeopardizes the inclusion of all eligible patients in the event of one single negative participation. It should also consider the possibility of a change of behavior because of the patient's awareness of their participation in a study, even if observational (Hawthorne effect).^27^

## Conclusion

In this study, we described the logistics and systematics of developing a clinical registry of STEMI patients in the digital platform REDCap, adapted from an existing clinical registry. This data may be useful for institutions planning to elaborate new registries or improve existing ones. The standardization of registry operation and the use of dedicated databases allow to optimize this tool in terms of quality and speed of implementation. The use of similar systems can also make sharing information across institutions easier as well as assist the development of new health technologies and in the decision making of public policies regarding cardiovascular disease.

**Figure 3:** – National Distribution - Centers of the multicenter Registry of Acute Myocardial Infarction. Via Google Drawings - https://docs.google.com/drawings
